# Does *Mycobacterium tuberculosis* var. *bovis* Survival in the Environment Confound Bovine Tuberculosis Control and Eradication? A Literature Review

**DOI:** 10.1155/2021/8812898

**Published:** 2021-02-05

**Authors:** Adrian R. Allen, Tom Ford, Robin A. Skuce

**Affiliations:** Agri-Food and Biosciences Institute, Veterinary Sciences Division, Bacteriology Branch, Stoney Road Stormont, Belfast BT4 3SD, Northern Ireland, UK

## Abstract

Bovine tuberculosis (bTB) is one of the globe's most common, multihost zoonoses and results in substantial socioeconomic costs for governments, farming industries, and tax payers. Despite decades of surveillance and research, surprisingly, little is known about the exact mechanisms of transmission. In particular, as a facultative intracellular pathogen, to what extent does survival of the causative agent, *Mycobacterium tuberculosis* var. *bovis* (*M*. *bovis*), in the environment constitute an epidemiological risk for livestock and wildlife? Due largely to the classical pathology of cattle cases, the received wisdom was that bTB was spread by direct inhalation and exchange of bioaerosols containing droplets laden with bacteria. Other members of the *Mycobacterium tuberculosis* complex (MTBC) exhibit differing host ranges, an apparent capacity to persist in environmental fomites, and they favour a range of different transmission routes. It is possible, therefore, that infection from environmental sources of *M*. *bovis* could be a disease transmission risk. Recent evidence from GPS-collared cattle and badgers in Britain and Ireland suggests that direct transmission by infectious droplets or aerosols may not be the main mechanism for interspecies transmission, raising the possibility of indirect transmission involving a contaminated, shared environment. The possibility that classical pulmonary TB can be simulated and recapitulated in laboratory animal models by ingestion of contaminated feed is a further intriguing indication of potential environmental risk. Livestock and wildlife are known to shed *M*. *bovis* onto pasture, soil, feedstuffs, water, and other fomites; field and laboratory studies have indicated that persistence is possible, but variable, under differing environmental conditions. Given the potential infection risk, it is timely to review the available evidence, experimental approaches, and methodologies that could be deployed to address this potential blind spot and control point. Although we focus on evidence from Western Europe, the concepts are widely applicable to other multihost bTB episystems.

## 1. Introduction

A comprehensive understanding of how transmission occurs is required in order to control the disease and to design and deploy effective control measures. Here, we focus on the bovine tuberculosis (bTB) episystems in Western Europe, but the concepts discussed are readily applicable to other jurisdictions. bTB eradication in Britain and Ireland is proving to be extremely difficult [1–4], contrasting with the better fortunes of other European countries [5]. The disease has a notoriously complex epidemiology worldwide, involving not only cattle but also multiple wildlife hosts. For example, in Britain and Ireland, a well-documented maintenance host is the European badger (*Meles meles*) [6]. The predominance of local risk factors in many epidemiological studies [7] and the geographic colocalisation of pathogen molecular types in cattle and badger hosts [8–10] are consistent with a shared epidemic; either these sympatric hosts transmit within and between themselves, to at least some extent, or both hosts are exposed to the same source, a component of which might involve a contaminated environment. In the UK and Ireland, there is an embedded cycle of infection, and there are probably embedded behaviours from the key players in the episystem. Local epidemiology is likely to differ by region and over time, so it would be unwise to generalise or extrapolate without comprehensive surveillance data.

In the recent past, as with human TB, it was largely assumed that the transmission of bTB between hosts was mostly facilitated by direct contact and inhalation of bacilli in relatively large (>5 *μ*M) “droplet nuclei,” causing the hallmark bTB lung pathology response [11]. However, more recently, a revival of the concept that a contaminated environment might be playing a significant role in disease epidemiology has occurred [5]. Contamination of the environment has been proposed to occur via the shedding of bacilli from infectious animals, a phenomenon well described in cattle [12], badgers [13], deer [14], and wild boar [15].

Indirect transmission from a contaminated environment has been hypothesised to involve generation of bacilli droplet nuclei from fomites such as soil, pasture, slurry, excreta, and the built environment either through host animal inhalation or ingestion [14, 16, 17]. A key component of the potential for a contaminated environment to contribute to bTB transmission dynamics is the survival of tubercle bacilli in these environmental matrices/fomites.

In the following, we review the evidence for environmental persistence of tuberculous bacilli which may constitute an infection risk and detail the methods and approaches that have been used to detect them and inform on the likely epidemiological risk.

## 2. Background: “First, Know Your Enemy…”—The Bacteria That Cause TB

### 2.1. Mycobacteria

Mycobacteria are common environmental microorganisms, but some species are significant human and animal pathogens. *Mycobacterium tuberculosis* is responsible for TB in humans [18]. *M*. *leprae* and *M*. *lepromatosis*, mycobacteria that cause leprosy, through a process of genome reduction, have become entirely dependent on the mammalian host for survival and dispersal. The recent recognition of potential reservoir hosts suggests that leprosy transmission dynamics may be more complex than had been appreciated [19, 20]. Most mycobacteria are found in soil or water where they occupy a variety of environmental and ecological niches: they are capable of advanced catabolism and degradation. Mycobacteria are routinely classified as rapid or slow growers, based on their *in vitro* growth rates [21]; the genetic factors that underpin growth rate differences and host adaptation are not well understood. Slow-growing species typically require more than 7 days for colonies to appear on solid selective media, while rapid-growing species form colonies within 2–5 days [21].

The slow-growing species tend to be more associated with an intracellular lifestyle and pathogenicity: rapid-growing species tend to be mostly environmental saprophytes and include only a limited number of opportunistic pathogens [22, 23]. Modern genomics suggests that slow-growing, pathogenic species likely evolved from rapid-growing, environmental saprophytes [24]. The availability of high-quality whole-genome sequences for an ever-expanding array of mycobacteria has allowed researchers to compare genomes of all major branches of the mycobacterial phylogenetic tree to identify genes enriched among rapid and slow growers. Results suggest that ancestral mycobacteria likely had a rapid-growth phenotype and that one major evolutionary separation into rapid- and slow-growing subgenera occurred. Furthermore, comparative genomics suggested that horizontal gene transfer, from nonmycobacterial genera, might have contributed to pathogenicity and host/niche adaptation, especially in the slow-growing species [24].

### 2.2. The *Mycobacterium tuberculosis* Complex (MTBC): Host Ranges and Potential for Environmental Persistence and Transmission

The globally important *M*. *tuberculosis* complex (MTBC) infects humans and animals causing devastating mortality and morbidity [25] and a spectrum of infections ranging from asymptomatic latent infection to active disease [26, 27]. The MTBC is a highly related group of pathogens that show marked host preferences; however, they are all now considered to belong to one “species,” although “species” is probably a redundant term for clonal bacterial pathogens [28].

The MTBC has recently been stratified, using molecular methods, into a number of very closely related lineages; lineages 1–7 comprise human-adapted lineages, whereas various animal-adapted pathogens (ecotypes) group together in a separate lineage [28]. *M*. *tuberculosis* var. bovis (*M*. *bovis*) [29] is host adapted to cattle and causes substantial economic losses as a barrier to trade locally, nationally, and internationally. It remains a significant zoonosis in many parts of the world [30, 31]. Four distinct global lineages of *M*. *bovis* have been described recently by phylogenetic analyses of ∼2,000 whole-genome sequences from multiple hosts, with lineages dispersed by location [29]. The origin of *M*. *bovis* is dated to East Africa ∼700–3,500 years BP, although the spatiotemporal distribution is complex; the impact of cattle import and export on pathogen and host distribution is clear. Within the wider mycobacterial family, the MTBC organisms are described as facultative intracellular pathogens, as opposed to obligate intracellular pathogens; they can survive and reproduce outside of the cells of those hosts they have evolved to infect [32]. The evidence we discuss below suggests that when they find themselves outside the infected host, or indeed between hosts, MTBC members, including *M*. *bovis*, are relatively resistant and resilient in the environment. They seem capable of relatively long-term survival on a wide range of matrices and substrates, including soils of wide pH range in experimental systems or under natural conditions.

Survival on various matrices is, however, only one part of the puzzle; how those surviving bacilli can then infect new hosts from those matrices is another. For years, the received wisdom was that these organisms infected hosts which were in direct, close respiratory contact. However, indirect mechanisms of transmission should not be excluded.

## 3. Direct or Indirect Transmission?

In host-pathogen systems where environmental transmission pathways may occur, complex interacting factors will influence pathogen transmission, including host susceptibility, environmental pathogen persistence, infectiousness and the mechanisms of host exposure, variables still largely unknown for many hosts, and MTBC bacteria. The *M*. *bovis* episystems seem particularly complex, with opportunities for direct and/or indirect transmission between and within livestock and wildlife hosts and potentially a contaminated, shared environment, depending on the mechanisms of excretion and contamination.

A number of pathogens, including some more associated with being transmitted directly, such as foot-and-mouth disease virus (FMDV), norovirus, and influenza virus, also use indirect transmission via a contaminated environment. Even for influenza, which has been intensely studied for decades, the relative importance of droplet and bioaerosol transmission and transmission from touching contaminated objects or surfaces is not clear. This seriously complicates and confounds disease epidemiology and control options, allowing spread from several nonhost sources [33]. An increasing awareness of the importance of direct versus indirect transmission in the context of the current COVID-19 pandemic has reenergised aerobiology and engineering research in this area.

Due to the multifactorial nature of bTB transmission dynamics, it is challenging to understand bTB epidemiology in host communities and the environment [15]. The well-documented limited sensitivity of ante- and postmortem diagnostic tests for bovine TB contributes to reduced prevalence ascertainment rates. However, there is renewed interest in trying to understand how environmental persistence might impact on MTBC transmission dynamics, particularly for *M*. *bovis*, where often, cryptic interspecies transmission at the interface between livestock and wildlife has confounded disease control in several episystems for many years. The distribution of the hallmark lesions (granuloma) in bTB-affected cattle supported the dominance of the respiratory tract as the major infection entry and exit route [34]. For *M*. *tuberculosis sensu stricto*, it has been suggested that TB is actually a disease of the lymphatic system, which merely uses the lungs as a point of entry and exit [35, 36]. The pulmonary entry route has been well characterised; the minimum dose required to establish infection was ∼1,000-fold less than that required to establish infection by ingestion. Pathological data seemed therefore more consistent with direct respiratory contact between hosts. While the latter remains an entirely plausible, though unquantified, hypothesis for intraspecies transmission, more recent observations of interspecies transmission suggest that it would be unwise to generalise about the principal routes used by the host-adapted members of the MTBC. Specifically, observational studies using cameras and proximity collar data from sympatric cattle and badgers in Britain and Ireland [37–39] revealed little, if any, direct contact between host species, thereby suggesting that some proportion of infection might be derived indirectly from the environment and that the preeminence of pulmonary lesions may be consistent with inhalation from both direct and indirect sources. While the respiratory system is the most likely point of entry and exit, the exact route taken, directly or indirectly from the environment, may vary.

Mounting evidence [40] suggests that different MTBC members favour different transmission routes, and this has significant implications for assessing the role of direct versus indirect transmission, the role of the environment, and how to develop effective interventions to interrupt transmission; hence, alternative routes may be relevant and underappreciated. There seem to be frequent opportunities for indirect badger-cattle contact, and environmental contamination might be a significant route of *M*. *bovis* transmission between badgers and cattle. If indirect transmission predominates, sympatric badgers and cattle may both transmit and acquire *M*. *bovis* infection via a shared contaminated environment. Potentially, some proportion of within-species transmission might also occur through an environmental route, depending on the mechanism [38].

In the following, we review evidence from multiple MTBC host-pathogen systems, including bTB in the UK and Ireland. These data add weight to the hypothesis that a contaminated environment may be playing an important and underappreciated epidemiological role in MTBC maintenance.

### 3.1. *M*. *tuberculosis sensu stricto* and *M*. *canettii*

There is now relatively high-quality scientific evidence, including studies using the guinea pig and mouse infection models, that supports environmental presence, persistence, and infectivity and a role for environmental contamination [41]. However, there is some uncertainty over the historical microbiology methods, results, and interpretation. For example, TB patient sputum was used instead of viable pure culture colony counts and pathogen detection, and identification was not as advanced as it is now. Consequently, it is timely to revisit and attempt to confirm or refute their interpretation using more modern methods and analyses.

*M*. *tuberculosis* causes an especially high rate of granuloma cavitation in human hosts and relies on generation of pathogen-laden particles for onward transmission. It may well have evolved pathogenesis mechanisms to promote cavitation directly, or indirectly via invoking a strong immune response [42]; these mechanisms may be associated with the so-called superspreading cases or events. Indeed, it has recently been shown that *M*. *tuberculosis* expression of the fatty acid sulfolipid SL-1 causes the host to cough, thereby promoting transmission [43, 44]. The TB bacteria that line the cavities are considered to be effectively a biofilm. Cavitation is a fundamental event in TB pathogenesis and epidemiology, a key driver of disease transmission that is not well studied or understood.

Although recently recreated landmark guinea pig experiments from the 1950s demonstrated that airborne-expelled droplet nuclei from infectious TB patients were the main route of transmission, they did not exclude a role for other transmission routes, including via the environment [45]. Both epidemiological and experimental evidence, including the use of more modern methods, now supports the presence of viable *M*. *tuberculosis* in many natural and built environments for periods up to several years after contamination [46]. Whether *M*. *tuberculosis* survival in the environment actually constitutes a significant risk to humans remains to be demonstrated empirically, but it does provide proof of principle that other host-adapted members of the MTBC might employ or even favour indirect transmission via the environment.

Researchers who demonstrated how *M*. *canettii* ingestion can recapitulate a pulmonary TB phenotype also tested whether *M*. *tuberculosis* could similarly infect experimental mice via ingestion. The received wisdom was that *M*. *tuberculosis* was exclusively transmitted by inhalation, whereas the highly related *M*. *bovis* and *M*. *canettii* were believed to be transmitted (in humans at least) by ingestion. In mice-fed *M*. *tuberculosis* Beijing genotype over a 28-day period, bacilli were detected in the lymph nodes and lungs of most subjects, suggesting that, after ingestion, *M*. *tuberculosis* was translocated to pulmonary tissue inducing a classical, respiratory disease phenotype [47].

*M*. *canettii* is a rare TB bacterium affecting humans that retains genetic traits of the proposed most recent common ancestor (MRCA) of the MTBC. It is believed to be poorly transmitted, if at all, by the inhalation route. To investigate whether *M*. *canettii* can infect hosts via the oral route, mice were fed 10^6^*M*. *canettii* mycobacteria and euthanised over a 4-week experiment. *M*. *canettii*-infected mice yielded granuloma-like lung lesions, and most mesenteric lymph nodes were polymerase chain reaction- (PCR-) positive soon after infection; most faeces were PCR-positive throughout. *M*. *canettii* seems to be readily translocated from ingestion to organs, including the lungs, making an environmental reservoir plausible [48].

### 3.2. *M*. *bovis*, Other MTBC Ecotypes, and Other Notable Veterinary Pathogens

In Michigan, evidence suggests that contamination of cattle feed via inadvertent supplemented feeding of wild bTB-infected deer, maintained for hunting, contributes to bTB incidence in cattle [49]. In experimental studies, calves exposed to feed used by *M*. *bovis*-infected white-tailed deer subsequently developed classical bTB. Similarly, ingestion may be driving bTB infection dynamics among deer, pigs, and possums in New Zealand or exposure to *M*. *bovis*-infected carcasses in the environment [50]. Ingestion of contaminated soil has been observed to result in the development of tuberculous granulomas in mice [51]. Epidemiological studies in Spain suggest that wild boar and red deer behaviour patterns and *M*. *bovis* environmental contamination may be contributing to observed transmission dynamics in this system [52, 53]. *M*. *bovis* DNA was detected in the environment at water holes, and DNA detection was correlated with the size of the water hole and the presence of susceptible animals [15].

*Mycobacterium mungi*, a relatively recently characterised member of the MTBC, has emerged as host adapted in wild banded mongoose in Northern Botswana, although it has yet to be successfully cultivated *in vitro* [40, 54]. Molecular examinations place the organism in wildlife-associated lineage 6 of the MTBC. Unlike *M*. *tuberculosis sensu stricto*, this pathogen does not appear to be transmitted primarily via an inhalation route or even by an oral ingestion route. It appears to transmit, between mongooses, via a novel mode of environmental infectious disease transmission, an environmental pathway where infected secretions, including urine and anal gland secretions, used in communication behaviours, infect the mongoose host through injuries/abrasions to the skin or nose. This would effectively circumvent natural social barriers, including territoriality, and allow between-group transmission, potentially without direct physical contact. Importantly, no environmental sources of *M*. *mungi* DNA have yet been found. These transmission observations potentially have epidemiological significance for other social species, including badgers [55].

In the UK and Ireland, the hypothesis of the preeminence of direct respiratory spread in close proximity hosts in shared airspace, resulting in the predominance of tuberculous lesions in the upper respiratory tracts of both hosts, has been challenged by proximity collar data [37, 38]. These observations have helped to revive the hypothesis that a contaminated environment may be playing some indirect role in inter- and intraspecies disease transmission; this hypothesis is not new. Previously, a role for badger excreta playing some role in contaminating pasture and feed [55] was proposed. The plausibility that both cattle and badgers could become infected by inhaling bacilli after inspecting contaminated, territorial urine trails or latrines has gained more attention of late [56]. The exact mechanism is unknown, but both hosts could conceivably create droplet nuclei via rumination and/or eructation. Renal pathology is the second most common pathology reported in infected badgers, and one previous study reported that badger urine contained 250,000–300,000 bacilli per ml [57]. In addition to urine deposited on pasture, badger faeces from latrines close to territorial boundaries may also act as potential environmental sources of *M*. *bovis* [56].

Other significant veterinary pathogens are transmissible from such inhalable environmental sources. *Coxiella burnetii*, the causative agent of Q fever, infects animals and humans exposed to contaminated wool [58]. Similarly, *Mycobacterium avium paratuberculosis* (MAP), which causes Johne's disease in ruminants, is reported to be rendered in dust derived from bovine faeces in animal housing and is a possible cause of infection [59]. Unlike *M*. *bovis*, transmission via ingestion from faecal shedding is the accepted main route, and the organism can survive for days to months in the environment, depending on the matrix [60]. In a study in the Netherlands, the environment was sampled following the introduction of two groups of cattle known to be shedding MAP. Bacterial DNA was detected at many sites within the housing, both before and after the introduction of MAP-excreting cattle [61], and was detected outside the barn in a pattern corresponding to the daily walking route of the farmer. No viable MAP were detected before the introduction of excreting cattle, but they were detected in the barn 3 weeks later at 7 of 49 sites and then outside the barn at 15 weeks. This illustrates the potential for widespread contamination of the internal and external farm environment, including the detection of viable bacteria in settled dust, which suggests potential for transmission via inhalation and possibly ingestion. In a subsequent study, such barns were sampled, with animals present, after destocking, after cold high-pressure washing, after having been kept empty for 2 weeks, and after the use of disinfectant [62]. MAP was detected by PCR in ∼80% of samples when animals were present. Viable MAP was detected in 6 of 9 samples and in 3 of 7 samples from different barns. Only 2 samples from each barn were positive for viable MAP after cold pressure washing, and no viable MAP was detectable if the barn was empty for 2 weeks or if additional disinfectant was used. No viable MAP was detected in any settled dust samples this time.

## 4. How Might the Environment Become Contaminated by *M*. *bovis* and Other Mycobacteria?

### 4.1. Cattle-Related Factors

Cattle may act to contaminate pasture and housing through shedding in excreta, respiratory droplets, and nasal mucus. Shedding in nasal mucus seems to be an extremely rare and intermittent occurrence; the stress of movement, testing, calving, etc., may play a role, but data are sparse [63]. BTB infection models confirm the shedding of organisms in early pathogenesis, with a potential for transmission and the failure of the tuberculin test to detect all infected cattle. BTB is primarily a respiratory infection; infectious particles may originate from sputum (the respiratory tract) or from contaminated fine dust particles, a potential environmental route [64, 65]. Onward transmission appears to require lesions in the lungs and associated lymph nodes, and contrary to dogma, most (40–73%) confirmed bTB reactors have lung lesions, although many are too small to be detected routinely at abattoir meat inspection [66].

While the generation of respiratory droplets may be the primary route by which cattle *M*. *bovis* contaminates the environment, it may not be the only one [67]. In Germany in 1955, Reuss [68] showed that *M*. *bovis* could be cultured from 10% of faecal samples from a herd that was mostly bTB positive. Within faeces, the bacteria stayed alive for at least 8 weeks. Typically, ∼10% of cattle with advanced bTB excreted bacteria in faeces [69]; however, the latter figure may well be conservative and could be as high as ∼80% [68–70]. Studies from Ireland suggested that up to 40% of infected cattle excrete *M*. *bovis* in faeces [71]. In 1959, Schneller [69] reviewed the potential risk of infecting cattle from pasture. In one such study, 23 of 42 uninfected cattle picked up bTB from a contaminated field. O'Reilly and Daborn [72] cited Schneller's findings [69], showing that, in graze plots experimentally irrigated with 10^2^–10^12^*M*. *bovis* per ml of water, 7, 14, and 21 days before permitting animal grazing, only 2 of 14 cattle that grazed the plot irrigated 7 days previously became infected. The remaining animals, grazing the pasture at other postbacillus irrigation time points, appeared uninfected. Schneller [69] concluded that the risk of rain washing bTB from infected fields to neighbouring fields was insignificant if washed out of the faeces and exposed to germicidal UV light. Also discussed was the practice of leaving fields empty after cattle grazed them for 12, 17, or 34 days [69].

*M*. *bovis* survival in faeces on pasture depends on available sunlight as well as any protection provided by the deposit. Faeces can remain infective for up to six months in winter but only 1-2 months in summer [73] before being biodegraded. Although herd hierarchies have been reported, cattle tend to avoid grazing close to cattle faeces initially, preferring to graze mature sward fertilized by faeces, making it unlikely that *M*. *bovis* infection is acquired directly from faeces deposited by grazing cattle. However, badgers regularly forage cattle faecal deposits for food [74]. This may provide an opportunity for transmission by ingestion, or possibly by inhalation, should aerosols with suitable properties be created.

#### 4.1.1. Mitigating Such Risk?

Solid manure (faeces) was not considered a major risk factor in spread, providing it was properly composted (for at least 30 days) before land spreading and did not produce droplet nuclei or other bioaerosols [75]. *M*. *bovis* survival in slurry can be observed up to 6 months/175 days [16, 75]. Consequently, manure and slurry should be stored at least 2 months and 6 months, respectively, to reduce risk of infectiousness. Previously, movement of slurry tankers within and between farm was found to be potentially risky for spreading infection over wider ranges [76]. More recent work from Ireland has indicated that grazing slurry-treated pasture, where slurry was stored for less than two months, was a risk factor for infection [71]. However, this risk factor was no longer significant in a subsequent multivariate analysis of persistent bTB breakdowns in Ireland [77]. A more recent NI-based risk factor study identified the use of contractors to spread slurry as a relative risk in bTB-affected herds [78] although the size effect was not large (OR: 2.83; 95% CI: 1.24, 6.49) compared to other badger-related and cattle movement metrics.

While not conclusive, the fact that some studies suggest a potential role for slurry-based contamination of the environment may be important; it is perhaps wise then to consider mitigation strategies. Chemical disinfection of slurry could be considered [79], with proper application of “thick lime milk,” likely to inactivate *M*. *bovis* within 24 h. Avoiding slurry application to grazing pasture may also be considered a useful intervention, instead applying it to arable land or grassland destined for silage. Creation of infectious aerosols by rain-gun slurry spreading is recognized as hazardous on farm and to contiguous farms. Such an application method can create aerosols which may be carried for several hundred metres [80]. Alternative slurry spreading practices, such as shallow injection or band spreading, are recommended to reduce the risk. While such “precautionary principle” mitigations would seem sensible, we were unable to find substantial published evidence that their application translated into measureable benefits on bTB incidence. As with potential biosecurity- and behaviour-based mitigations, sufficiently powered, cost-effective, and robust studies, with a measurable benefit on bTB prevalence, have not yet been reported.

The relative importance of spread of *M*. *bovis* in slurry and faeces via the environment is unknown, but many studies converge on untreated contaminated cattle slurry and manure as a potential risk [81, 82] for spread by the respiratory and/or ingestion routes. The importance of this route is determined by many factors which are difficult to observe and quantify. At least one infected bovine per herd must be shedding *M*. *bovis* in faeces, urine (unlikely), or milk disposed in slurry, and an infectious dose must come into contact with at least one susceptible cattle host; the pathogen must survive storage, substantial dilution, and the aerial or ground environment for long enough to contact a susceptible host. To add to the lack of empirical evidence, an exact mechanism for generating infectious particles from contaminated environmental matrices has yet to be demonstrated. Having perfect knowledge of all these details is impossible within the field setting, but demonstrating viability of organisms *in vitro* and highlighting potential mechanisms for transmission could help propose and evaluate more rational interventions, raising the need for empirical research that could more fully inform these issues.

### 4.2. Wildlife-Related Factors

The potential role of infectious wildlife in any environmental contamination needs to be considered. Badgers are an accepted wildlife reservoir for *M*. *bovis*, in the UK and Ireland at least. The distribution of infection in naturally infected badgers sampled in Ireland has been assessed using detailed pathological and microbiological techniques [13]. BTB was confirmed in 43.2% of culled badgers, taking ∼32 samples per badger. About 50% of bTB-positive badgers had no visible lesions; infection was well dispersed in badger carcasses. The main sites were the lungs and ancillary lymph nodes, suggesting that infection via the lower respiratory tract was the most likely transmission route. As with cattle, this raises the possibility that badgers could be contaminating the environment via respiratory exudates and mucus. However, studies from the 1970s in Britain, and more recently, the randomised badger culling trial (RBCT), also revealed that a common site for tuberculous lesions in badger carcasses was the renal system [83–85]. Badgers do leave urine trails on pasture, and bacilli can be cultured from urine. Bacilli excreted in badger urine have been observed to survive on pasture for ∼3 days in summer and ∼14 days in winter, with the differing seasonal outcomes thought to relate to relative intensity of solar UVA radiation [86]. It is therefore not surprising that this would be a potential source of environmental contamination with *M*. *bovis* and a means by which badgers could indirectly infect cattle.

Alongside potentially infectious urine deposits, another known part of badger social behaviour and ecology is the digging of faecal latrines at territorial boundaries. Culture and molecular detection [56] of *M*. *bovis* from faeces confirms their potential role in further contaminating the environment. Research by the University of Warwick used molecular direct detection methods to index and quantify locations where bacilli were shed in faeces. In these studies, potential badger population infection hotspots were investigated, and a proxy measure for bacterial load and shedding using quantitative PCR (qPCR) was deployed [87]. Infected badgers were observed to shed between 1 × 10^3^ and 4 × 10^5^*M*. *bovis* cells per g faeces, potentially creating a significant and variable environmental reservoir [56]. Culture from faeces is notoriously insensitive, but the study's qPCR method detected up to 43% infectivity in some well-studied badger social groups in Woodchester Park, Gloucestershire, UK [87]. An important caveat here, however, is that culture is an important indicator of cell viability in a way that many molecular tests are not; they tend not to distinguish live from dead bacterial cells.

A further relevant aspect of badger ecology, and of mustelids more generally, is the scent marking employed to mark territory via anal and caudal glands [88]. It is currently unclear whether badger scent marking contributes to environmental contamination, similar to that of *M*. *mungi* in the mongoose, but it may be worth investigating whether this could be another source of *M*. *bovis* transmission both within and between species.

### 4.3. Other Environmental Hosts: Wildlife and Companion Animals

Although many animals are susceptible, relatively little is known about the status and potential of other wildlife in the UK to contribute significantly to *M*. *bovis* environmental persistence and spread [89]. In a badger proximity and farm surveillance study in Northern Ireland [37], cats were frequent visitors to farmyards. Farm and feral cats would be considered as spillover, potentially sentinel hosts for bTB in cattle, but reports of culture-confirmed *M*. *bovis* in cats are relatively rare and mainly in endemic areas. There are a few publications on bTB in farm cats but apparently none on feral cats. Clinical signs in affected cats may include enlarged lymph nodes, infected bite wounds, poorly healing ulcers, especially around the head, and poor body condition. A cluster of cat cases in Berkshire, UK, around 2012 suggested exposure to the same source, but it was not possible to exclude cat-to-cat transmission [90].

It has been suggested that cats might be exposed by contact with infectious cattle in the farmed environment, for example, where there is bTB mastitis, cats might become infected via ingestion of contaminated raw milk. That said, bTB mastitis in cattle is rare these days [91]. Cats might also be exposed directly or indirectly if exploring the farm or wider environment or badger setts. Small rodents have also been observed to frequent badger setts and would be a common feature of the farmed and built environment. The relatively recent recognition of “bite and fight” wounds around the heads of affected cats has led to the intriguing suggestion that, either, these cats have been exposed and infected directly by affected rodents, or the exposed bite and fight wounds have been infected and contaminated by the environment [92]. Direct contact between cats and badgers seems unlikely.

There are very few reports of confirmed *M*. *bovis* in dogs in the UK and Ireland, suggesting that dogs are not particularly susceptible and only in exceptional circumstances. A high-profile recent outbreak was reported in a hunting kennel, resulting in the voluntary testing and euthanasia of 97 foxhounds. Investigation centred on the feeding of contaminated raw offal derived from fallen livestock. Dogs are not considered to play a significant role in bTB transmission in the UK [93].

There is little evidence of these companion animals shedding bacilli to the environment, but the possibility cannot be excluded.

### 4.4. Invertebrate Hosts

It is becoming apparent that invertebrate hosts in the shared farmed environment might act as vectors for *M*. *bovis* and might play some role in dissemination and persistence. Earthworms have been shown to ingest *M*. *bovis* from cattle faeces and to shed the bacilli in their castings [94]. It is noteworthy that, across Britain and Ireland, earthworms comprise a substantial component of the diet of badgers [95–97] and that Britain and Ireland have some of the highest species diversities and densities of these important ecosystem engineers [98, 99].

Similarly, environmental single-celled organisms, such as free-living amoebae (FLA), have long been hypothesised to be a “nursery” for intracellular bacteria within the wider environment [5], perhaps even having been the hosts in which nonpathogenic bacteria transitioned to become pathogens [100]. Recent experimental evidence confirms that survival within FLA hosts occurs [101], using the same mechanisms employed to persist in vertebrate macrophages. Furthermore, exposure of laboratory animals to infected FLA can lead to transmission of pulmonary TB [102]. However, some studies with other FLA species have concluded differently. In a study attempting to detect internalised mycobacteria by sampling faeces at badger latrine, samples were culture and PCR negative. Experimental coculturing of the FLA *Acanthamoeba castellanii* with virulent *M*. *bovis* led to a significant drop in *M*. *bovis* titre, suggesting that at least some FLA may actually suppress long-term environmental persistence as opposed to facilitating it [103].

Arthropod (ecto)parasites are rarely reported on UK and Ireland badgers. Consequently, they would appear to be unlikely vectors of transmission between badgers and cattle [104]. Interestingly, an epidemiological association between tick-borne encephalitis, immune suppression, and human TB has been reported [105]. *M*. *bovis* has been reported in ticks taken from the skin of infected hosts; studies in Armenia suggested that ticks may carry viable mycobacteria for months [16, 106]. It is possible, in theory, for ticks to transmit *M*. *bovis* between cattle and wildlife, although no published evidence suggests that modifications to animal husbandry would actually help in a measureable way [16].

Intriguingly, the obligate intracellular pathogen *M*. *leprae*, which causes highly debilitating leprosy in humans, has never been cultured *in vitro*; it can be cultivated *in vivo* in experimental animals. It was mostly believed to be transmitted by close direct contact. However, recent research has suggested that sources of infection and routes of transmission may be significantly more complex and may include indirect transmission via wildlife reservoirs or invertebrate vectors, such as FLA [107, 108]. Surprisingly, because leprosy and *M*. *leprae* were believed to be eradicated from Great Britain (GB), *M*. *leprae* and the related *M*. *lepromatosis* were detected recently in red squirrels in GB. Similarly, the transmission of Buruli ulcer, caused by infection with *M*. *ulcerans*, remains an enigma. However, recent experimental infections with mice indicate a possible role for mosquito (*Aedes notoscriptus* and *Aedes aegypti*) blood feeding in the transmission of this neglected tropical disease [109].

These findings indicate that our sole focus on livestock and wildlife hosts such as deer, badgers, wild boar, and possums may be underappreciating an important source of epidemiological risk and that a wider “ecosystem health” approach to bTB may be more appropriate. The complex cycling of intracellular pathogens between these nonbovine vertebrate and invertebrate reservoir hosts and the environment may also warrant further research.

## 5. Bioaerosols and Droplet Nuclei?

In human TB epidemiology and pathogenesis, at least, there is probably a need to make a clearer distinction between true bioaerosol transmission and that of the so-called “droplet nuclei,” containing expelled TB bacteria; there is considerable uncertainty and ambiguity in the literature. The term “aerosol” tends to be used interchangeably with “droplet nuclei” when they are not quite the same thing, and there may be important implications for transmission and control. The importance of clarifying such ambiguity is illustrated clearly in the current COVID-19 pandemic.

In general, aerosols refer to the very small droplet sizes generated (<3 *μ*M), whereas droplets tend to refer to larger particle sizes, which drop to the ground relatively quickly under gravity before they evaporate to cause local contamination [110]. Transmission through large droplets is “droplet or contact spread”; transmission occurs by touching a surface contaminated by droplets or by exposure within, for example, 2-3 m speaking distance when an infectious host is excreting. Aerosols are much smaller droplets and overcome gravity to suspend in the air for long periods of time. They can evaporate before landing to leave the aerosol particles able to float relatively long distances, referred to as true “airborne” transmission. Consequently, an infectious aerosol is a collection of pathogen-laden particles suspended in air; particles may be inhaled by (or deposited on) a susceptible person or deposited on a surface. Such transmission is biologically plausible when aerosols are expelled by an infectious host, the pathogen remains viable in the environment for some period of time, and target host organs and tissues are accessible to the aerosol [111]. Time in contact with an infectious case is also an important variable. To recapitulate the hallmark pulmonary pathology seen in cattle cases of bTB, it seems pertinent to speculate that an environmental source of infection would need to generate some form of infectious bioaerosol. However, exact mechanisms that can achieve this are unknown. Animal inspection of infectious environmental fomites may be one potential mechanism. However, generation of aerosols after ingestion, especially in ruminants, should not be discounted.

Improved understanding of aerosol science should allow rational explanation and intervention selection for infectious diseases. Mycobacteria can be shielded from environmental stresses in multibacillary aggregates generated from some hosts and environments; this improves their resilience further and suggests short distance transmission between close-contact predominates [112].

## 6. How Long Can MTBC Persist in the Environment?

A prerequisite for the efficacy of indirect transmission from the environment is the longevity of pathogen survival in environmental matrices and fomites. Recently, the relevance of MTBC members' persistence in a variety of environmental matrices has been revisited. While the potential role of the environment in bTB epidemiology is plausible and a reemerging area of research with more modern methods, classical epidemiology studies, which investigated bTB risk pre- and post-FMD restocking, concluded that cattle-cattle transmission was still potentially the most important source. However, there was an associated “stationary” breakdown risk for bTB persistence on the farm, outside of cattle, which decayed with “time since last breakdown.” Further cohort studies of continuously stocked and restocked herds following FMD indicated that an observed lower risk of herd breakdown in the first year after restocking might be due to a temporary reduction in the force (load) of infection on the farm. However, this reduction did not persist following the (re)introduction of cattle, suggesting that cattle themselves were contributing significantly to the observed persistence [113]. The observation that cattle-to-cattle transmission may be more important in overall disease dynamics does not preclude that a contaminated environment may still play an important role in between- and within-host transmission. In the following, we review the literature to build a consensus on the significance and longevity of *M*. *bovis* persistence in the environment.

### 6.1. Cattle Manure/Slurry

Studying *M*. *bovis* in the environment is challenging; studies have tended to simulate the environment by artificially spiking samples. Using these types of methods, *M*. *bovis* has been shown to survive in stored slurry for up to 6 months, but on pasture up to 2 months in summer and up to 6 months in winter [114]. The pathogen survives better in cool, damp, and dark conditions. Microbial fermentation and metabolism in properly composted solid cattle manure should achieve sustained temperatures >50°C, which should kill *M*. *bovis*, although it might survive in some parts of the manure [114]. Slurry would be expected to present a higher risk [76]. One could argue that a component of such exposure represents indirect infection (inhalation/ingestion) by the faecal-oral route.

No direct studies have been carried out to investigate *M*. *bovis* survival through the anaerobic digestion (AD) process, which is gaining traction for management of farm/other waste. Thermophilic ADs (>50°C) should, hypothetically, neutralise microbes more efficiently than mesophilic ADs (20°–30°C) [114]. Research on the related pathogen, *M*. *a*. *paratuberculosis*, showed survival for up to 2 months in mesophilic ADs. To date, it is unclear to what extent the products of AD can be rendered safe and much depends on the survival structures (such as sporulation) and strategies (such as dormancy) used by different pathogens [115].

### 6.2. Silage, Forage/Foodstuffs, Soil, Pasture, and Water

Contaminated silage is another potential environmental source, although the ensiling process should reduce available oxygen, reduce pH, and raise the temperature (20°–30°C). However, pH 4-5 and temperatures obtained are well tolerated by *M*. *bovis*. The use of a silage clamp is associated with elevated risk in some risk factor studies. There are limited data on *M*. *bovis* survival in silages. However, recent US research [116] investigated whether *M*. *bovis* remained viable in ensiled forages (alfalfa, mixed grasses, and corn silages). Previous US spiking and direct detection work in NE Michigan [117] indicated that *M*. *bovis* could survive for at least 16 weeks on common animal feedstuffs, which can be available to white-tailed deer; feed contaminated by infected deer may be one such spillover transmission route back to cattle.

A field experiment in Michigan revealed that *M*. *bovis* survived significantly longer in cooler seasons than in spring or summer: supplementary winter feed was thought to become contaminated by infected deer shedding *M*. *bovis* in various discharges [118]. *M*. *bovis* survived at all temperatures on all feed for at least 7 days and at 23°C. *M*. *bovis* was isolated from feed (apples, corn, and potatoes) at 112 days. *M*. *bovis* was isolated from inoculated substrates up to 88 days in soil, 58 days in water, 58 days in hay, and 43 days on corn [117]. *M*. *bovis* may persist long enough to be a risk to deer and cattle in this episystem. Over ten sampling days, to simulate ensiling, six replicate feedstuff samples were vacuum packed in film pouches; four were spiked with viable *M*. *bovis*, and two were nonspiked controls; pouches were stored in the dark at room temperature and analysed on designated days using culture (viability) and PCR. *M*. *bovis* was not cultured from alfalfa or corn silage after two days but was cultured from mixed grass silage for twenty-eight days after inoculation/ensiling. *M*. *bovis* DNA was detectable in samples of all feedstuffs for the duration (112 days). The PCR tests provided a proxy estimate of DNA concentration or genome equivalents; no significant DNA degradation was detected across the study. No controls were positive for *M*. *bovis* by culture or PCR tests. Consequently, properly ensiled forages seem unlikely sources for *M*. *bovis* transmission, although further research was indicated to investigate whether ensiling actually kills *M*. *bovis* or drives it to transition to a dormant, nonreplicating state. If so, what environmental conditions then render it infectious in the environment? Therefore, currently, *M*. *bovis* survival in silage cannot be excluded.

Cattle faeces, containing *M*. *bovis*, were not infectious to guinea pigs once ensiled with grass for ten weeks in a mini silo; lack of oxygen and acidity may have reduced the infectivity. The acidity of silage declines to approximately pH 4.0, although *M*. *bovis* can survive for 20 days at pH 4-5 in yoghurt [119]. Temperature during ensiling and storage increases to ∼30^o^C, which is close to optimal for *M*. *bovis* growth. Maize silage is no less/more likely to maintain *M*. *bovis*, and maize cobs are particularly palatable to badgers. Circumstantial evidence suggests that maize can be contaminated by diseased badgers that contaminate the silage clamp directly or indirectly. Currently, silage cannot be excluded as a risk, and steps should be taken to avoid contaminating silage fields with slurry. Consequently, badgers should be kept away from silage pits, and clamps, particularly maize silage [16]. Some bacterial pathogens of cattle, such as *Listeria monocytogenes*, streptococci, and enterococci, have been shown experimentally to survive the ensiling process. Potential persistence of *M*. *avium paratuberculosis* (MAP) was investigated by spiking feedstuffs and using PCR methods; MAP DNA was detected after ensiling. MAP culture was either not attempted or was not successful. The potential for MAP to remain viable and perhaps infectious in ensiled feedstuffs remains uncertain [120].

In an Australian mid-1980s study, *M*. *bovis* survived (culture positive) for 4 weeks in nonsterile dry and damp soils in 80% shade, in darkness, and in the laboratory [121]. *M*. *bovis* was not reisolated at 4, 8, or 32 weeks from any matrix exposed to sunlight or from faeces under any conditions. A number of more recent studies, in different countries, suggest that *M*. *bovis* survival in environmental matrices is variable [122], with bacilli in faeces or faeces-contaminated soil appearing to remain viable for up to six months in some studies [123]. Soil systems seeded with *M*. *bovis* and incubated at 4°C and 22°C exhibited greater survival at the cooler temperature; results for differing soil types were inconclusive [124].

Prevailing weather is likely to dictate the viability of *M*. *bovis* bacilli on pasture when uninfected cattle graze after infected cattle. *M*. *bovis* is susceptible to germicidal UV irradiation provided by sunlight within ∼12 h duration. In dull, warm, and wet weather, contaminated pasture can transmit infection several weeks after grazing by clinically infected cattle (reviewed in [16]). One week after resting pasture following grazing by infected cows, there was ∼6% daily chance of a noninfected cow acquiring infection, reducing to 2% daily after two weeks of rest. These experiments are quite old, methodology has improved, and mesenteric involvement and generalized bTB are allegedly rare nowadays.

Although historically not considered relatively high risk, cattle-cattle transmission has been demonstrated experimentally outdoors at pasture. However, the mechanism of transmission (whether direct or indirect or some combination) was not clear. Stocking density influenced the probability of transmission at pasture; reducing it should proportionately reduce the probability that cattle contact contaminated grass before any bacteria are destroyed by germicidal sunlight. Soil can be ingested by cattle grazing pasture, comprising ∼5–10% of the fresh weight intake and 10–15% of the dry weight intake. The movement of soil-contaminated fodder between farms may also be risky [16]. Cattle tend to consume soil to offset mineral deficiencies and for behavioural head rubbing, during which they create dust and potentially pathogen-laden particles. Relatively more soil would be ingested when pasture sward is short, and soil may also contaminate silage. Providing cattle with field-based mineral supplements may reduce the attractiveness of the soil.

Maddock [125] showed that calves could be artificially infected via ingestion of high doses of an *M*. *bovis* emulsion; however, no acid-fast bacteria were seen in their faeces. These calves were then dosed with infected whey until they excreted *M*. *bovis* in their faeces; at postmortem inspection, they showed no obvious kidney or mesenteric involvement. Excreting calves were grazed for three weeks following which 2 uninfected calves were introduced to graze for 3 weeks on one of three plots at intervals following removal of the original calves. No signs of bTB infection were evident in any of these calves postmortem. In a further experiment [125], a cow with bTB mastitis and excreting *M*. *bovis* in her faeces was grazed for 9.5 weeks. Naïve calves were introduced to contaminated plots at monthly intervals. Again, no infection was demonstrated. Consequently, there appears to be no real consensus in the literature about the risk posed by grazing cattle on fields that previously contained bTB reactors.

*M*. *bovis* survives in running water and when cultured in buffered saline and egg-based media for 300–400 days and >6 years, respectively [16]. Running water could also be directly contaminated with cattle or wildlife excreta, although an Irish study has shown that availability of natural water was not a significant risk factor [77]. Water troughs may however become contaminated with *M*. *bovis* from cattle sputum or from wildlife and thereby constitute a transmission risk. Regular cleaning and disinfection and avoidance of stagnation are advisable, especially if bTB reactors are detected. Where there are signs of badger activity, it would be advisable to prevent badgers from accessing cattle water troughs, by raising them to >80 cm.

A study of African buffaloes, the maintenance host for *M*. *bovis* in Kruger National Park, investigated whether shedding of *M*. *bovis* in nasal and oral secretions might lead to contamination of ground or surface water and subsequent transmission to other species [126]. *M*. *bovis* was not detected (cultured) in trough water, suggesting that diseased buffalo does not commonly shed the organism in detectable quantities in nasal or oral discharges. Surface water was considered unlikely to be significant in the transmission of bTB in this free-ranging episystem.

## 7. Methods for Investigating *M*. *bovis* Persistence in Environmental Matrices

An important and necessary next step towards hypothesis-driven research on environmental persistence of *M*. *bovis* is the development of suitable scientific methods and models. This is an emerging research area, and as such, there are limited empirical data. While there is some crossover, in general, the existing methods tend to fall into two broad categories:

1. Culture- and molecular-based (culture-dependent and culture-independent methods, respectively) methods for detecting *M*. *bovis* in “real-world” field samples.
2. Establishment of *in vitro* environmental micro- and/or mesocosms to which *M*. *bovis* can be introduced/spiked, potentially alongside vertebrate and/or invertebrate host organisms. Simulated environmental conditions can be varied, and molecular and culture methods can be used to assess effects on pathogen persistence.

In the following, we review the literature pertaining to these methods and identify potential difficulties/limitations from both a methodological and inferential standpoint. Investigations into environmental persistence and transmission are hampered by the lack of standardised and validated methods, whose performance characteristics are understood for reliably detecting bacilli in different matrices and fomites.

### 7.1. Real-World Sampling and Direct Pathogen Detection

The general principle of applying molecular- and culture-based methods to real-world samples to detect pathogens or other environmental bacteria has an acceptable pedigree. A considerable amount of work on developing direct detection methodologies for the human pathogen, *M*. *tuberculosis*, has been undertaken using various clinical samples. Konno et al. [127] applied mycobacterial growth indicator tube (MGIT) culture and real-time PCR (RT-PCR) methods to detect *M*. *tuberculosis* in elderly human stool samples. Similarly, the World Health Organisation (WHO) has recently endorsed the semiautomated PCR test GeneXpert® MTB/RIF (Cepheid Corp.) as the frontline test for presumptive human TB in a range of clinical samples, most notably sputum [128]. It is telling, however, that while there has been an understandable drive to develop rapid diagnostics with better performance characteristics, progress and validation have been disappointing—“*rapid tools but slow progress*” [128]. Two problems loom large on the TB direct detection horizon. Culture methods, in general, lack sensitivity [129], and molecular bacteriology detection methodologies have had limited application and success, having tended to be variable and poorly reproducible, unlike the well-documented success achieved with viral load testing. Studies have tended to use general, but not standardised, methods to be small and underpowered or not adequately controlled, and methods have not been widely used for environmental detections. Sampling and study design are important, and it would not be trivial to validate such methods before deployment in higher-throughput studies.

The direct detection “issues” mentioned above hold true for detection of other members of the MTBC and related mycobacteria, including *M*. *bovis*. To set the scene, we consider a recent study that attempted to detect general environmental mycobacteria from a wide range of regions in Ethiopia [130]. Generic molecular methods (oligotyping) identified MTBC in >90% of water samples; mycobacteria implicated in lower BCG vaccine efficiency were detected in both soil and water. *M*. *bovis* was detected in water at up to 10^2^ genome equivalents per ml. Environmental predictors of both soil and water *Mycobacterium* communities were identified. The methods used, as with other studies, are not standardised or “validated,” with DNA extraction from matrices undertaken using general methods and detection of mycobacteria using a variety of PCR primers targeting some markers which are species-specific and some which are not—mpb64, mpb70, hsp65, 16S rRNA genes, or IS*6110* insertion sequences [130–135]. The latter is an important point, which we return to. Deploying species-specific primers is crucial to making informed inferences about survival of *M*. *bovis*.

An additional, significant caveat in all molecular-based *M*. *bovis* detection methods is the capacity to determine whether the bacilli detected are actually viable. *M*. *bovis* can be quiescent (viable, but nonculturable) under some conditions. Such information is obviously of crucial epidemiological importance. Detection of genomic DNA can be observed for living and dead bacteria, and *M*. *bovis* genomic DNA has been observed to survive in soil for up to 21 months [131]. Some groups have attempted to address this difficulty by using methods which target short-lived RNA molecules associated with cellular activity/viability—specifically, 16S rRNA. Similarly, 16S rRNA has been used as a target for TB diagnosis in tissues in the mycobacterial load assay (MBLA) [136]. It is perhaps better to use additional culture alongside molecular methods to definitively confirm viability, although even this has well-documented sensitivity limitations.

Next, we review the methods and variations used by several international groups in their application of the basic molecular detection methodology, in pursuit of developing *M*. *bovis*-/MTBC-specific tests for detecting bacilli in the environment.

#### 7.1.1. United Kingdom and the Republic of Ireland

Researchers from Warwick University and Ireland used direct detection molecular methods to propose that *M*. *bovis* DNA could persist in environmental samples as potential routes of transmission [137]. Specifically, the researchers used a qPCR method which targeted a specific sequence polymorphism in the *M*. *bovis* genome. A variety of such sequences have been used to detect MTBC, but the Warwick team settled on indexing the *M*. *bovis*-specific RD4 deletion. The assay was applied to soil samples which were processed for whole community nucleic acid extraction; *M*. *bovis* DNA is not believed to persist for long in the environment (<10 days). Consequently, a molecular detection signal suggests the presence of live (or recently dead) *M*. *bovis*. These methods were not initially designed to distinguish live from dead bacteria and, before application to field samples, were tested in BCG-spiked *in vitro* soil samples (see below). The PCR method detected *M*. *bovis* in environmental samples taken from a bTB-affected farm at 4 and 21 months after contamination, with a range 1 × 10^3^–3.6 × 10^3^ genome copies per gram of soil, depending on the sampling area. Samples taken around badger setts showed high levels of *M*. *bovis* DNA persistence, providing evidence that *M*. *bovis* DNA could persist in the farmed environment and that climatic factors might influence survival. A follow-up DEFRA study suggested that this PCR test had a sensitivity of 98% and specificity of 97% and, consequently, would produce both excessive false positives and negatives, making interpretation challenging.

In theory, such PCR tests could be used to attempt to detect infected badger groups from badger faeces sampled at latrines. However, since not all group members would be infected and not all infected would be shedding, this requires extensive and resource-intensive repeat sampling. DEFRA recently have allowed private use of additional nonvalidated tests, such as PCR, but at the herd keepers' own risk and expense. Potentially, such PCR tests could be used to attempt detection in various matrices and environments on and around farms. The test performance characteristics need to be considered, and sampling frames need to be designed accordingly. However, despite detecting pathogen DNA, whether *M*. *bovis* can sporadically or sustainably be rendered into an infectious form from these environmental matrices remains elusive. No evidence was provided to indicate that environmental detection by PCR had any predictive epidemiological value in local cattle herds [137]. At that time (2010), DEFRA ruled that such PCR tests were not fit for this purpose [138] and recommended rigorous validation and interlaboratory ring trials.

Subsequently, a validation ring trial of the qPCR test was reported [139]; three laboratories from two different countries participated. Sample panels comprised negative badger faeces spiked with a dilution series of *M*. *bovis* BCG Pasteur and field samples of badger faeces of unknown infection status from badger latrines in both high and low bTB incidence in local cattle. There was minor interoperator and interlaboratory variation, but good concordance between the three laboratories. There was good agreement in test positivity, especially at spiked levels of 10^5^ cells per gram or above. With latrine faeces, the PCR test showed high reproducibility despite low numbers of samples being test positive in any laboratory. There was negligible PCR inhibition reported due to sample matrices.

Despite encouraging results using the same sample panels, the issue remained that detecting *M*. *bovis* at specific on-farm locations was difficult without extensive replicate sampling. The need for well-designed field studies was evident. Consequently, the performance of the qPCR test was assessed in the well-studied Woodchester Park ecosystem in Gloucestershire [87]. Metadata (badger IGRA, Stat-pak, culture) have been collected on this high-density, undisturbed badger ecosystem for several decades. This allowed the qPCR results to be compared to other animal- and social group-level data, such as immunoassay results. The number of faecal samples required per zone varied between 5 and 50, depending on incidence. They concluded that qPCR testing of badger latrine faeces was likely to be as sensitive, if not more so, than live trap sampling in detecting *M*. *bovis* in badger populations. Results using this qPCR test correlated strongly with immunoassays applied at the social group level. Further field studies, using these sampling and detection methods, identified potential infection hotspots in the badger population and quantified the variability in *M*. *bovis* load. Infected badgers were detected as shedding between 10^3^ and 10^5^*M*. *bovis* cells per gram faeces, creating a substantial and seasonally variable potential environmental reservoir [56]. This molecular system has since been applied in several studies to test environmental substrates such as soil, badger faeces, and cow slurry [56, 87, 130, 135]. In Northern Ireland, the use of immunomagnetic separation techniques, to capture and enrich bacilli from diagnostic and environmental samples prior to confirmation by PCR and/or culture, has been demonstrated [140]. However, the technique, when applied as a lateral flow assay, detected only high numbers of shed *M*. *bovis* in badger faeces [141].

In GB, DEFRA has recently allowed the application of additional, nonvalidated tests at the herd keepers' own risk and expense. One such test uses a generalist bacteriophage and specific PCR or isothermal amplification to detect viable *M*. *bovis* in bovine blood [142]. This test has not yet been adapted, evaluated, or calibrated in a gold standard test using environmental samples where it may have additional utility.

#### 7.1.2. Continental Europe

In Spain, Gortazar and colleagues used molecular detection methods, together with other metadata, to investigate multihost systems [135, 143]. Through live sampling of wildlife (wild boar and deer) and their environments, they showed through qPCR, culture, and most probable number (MPN) statistical methods that compared to wild boar, red deer presented a higher risk of being supershedders in that episystem.

The same Spanish group proposed that indirect transmission might be facilitated if MTBC bacilli persisted in the environment sufficiently long to provide an exposure risk to sympatric domestic and wild animals [15]. They investigated environmental persistence and the use of water resources in two bTB endemic areas in southern Spain using camera traps and a new MTBC‐specific PCR on samples taken at watering sites. Just above half (55.8%) of the watering points tested PCR positive for MTBC in shore mud samples; 8.9% of their water samples were PCR positive. Their results help to understand the potential role of indirect routes of cross‐species bTB transmission. They highlight the importance of environmental features in maintaining infection in multihost episystems and help to better target control strategies for bTB at the wildlife/livestock interface.

The methods this group employed were a simple DNA extraction using the phenol/chloroform solvent extraction protocol and the IS*6110* PCR. Both methods are standard but difficult and multistage. The IS*6110* insertion sequence is generally believed to be a good diagnostic marker for members of the MTBC, having been the marker of choice for molecular detection and genotyping of *M*. *tuberculosis* in humans for many years [144]. However, IS*6110* detection is not able to distinguish between *M*. *tuberculosis* and *M*. *bovis*. In addition, homologues of IS*6110* may be present in other nontuberculous mycobacteria [145]; hence, the specificity of this marker may be suboptimal and could lead to misidentification in environmental matrices.

More recently, the Gortazar group advocated the use of environmental DNA sampling and interpretation for bTB risk assessment in multihost episystems [146]. In a bTB hotspot, environmental MTBC DNA was detected on 12 of 24 cattle farms sampled (50% positive farms; 31.3% positive samples). Environmental MTBC DNA detection also indicated significant differences in the use of remote grazing and in woodland proximity by infected animals, suggesting that animal-side, environmental DNA sampling may be useful in herd-level contact risk assessment. However, there was no significant association between the higher-risk area and the detection of environmental DNA. This study is interesting because, for the first time, environmental DNA detection was deployed systematically as a proxy for herd-level exposure risk. Culture and molecular detection methods were discussed and looked to be standard and repeatable. Of interest are the novel bTB DNA detection methods. Detecting MTBC DNA on cattle hide suggested ongoing contact with a contaminated source, whether other cattle, wildlife, or the environment more directly. Low bTB prevalence and detection of random test positives suggested an environmental origin for new cases. Although the study was unable to identify the origin of bTB infection in the herds studied, it did help identify additional risk factors that could possibly be mitigated via farm biosecurity. The authors considered that soil sampling might be a valuable complementary tool to provide a more complete epidemiological picture.

Contemporaneously to these Spanish groups, French laboratories have also been exploring *M*. *bovis* persistence in environmental substrates. Barbier et al. [147] applied these methods to matrices gathered from the Côte-d'Or region. They used molecular methods based on qPCR (double fluorescent dye-labelled probe) to monitor MTBC occurrence or *M*. *bovis* in environmental samples (including spring water, sediments, and soils from badger sett entrances) collected at pasture where infected cattle and wildlife were reported. Three qPCR assays were developed to target the MTBC (IS1561 and Rv3866 loci) or *M*. *bovis* (RD4) and validated using *M*. *bovis* spiked soil, water, and faeces (see below). MTBC and *M*. *bovis* were detected in environmental samples from bTB-affected farms at up to 8·7 × 10^3^ genome copies per gram of badger sett soil. Results suggested that *M*. *bovis* was detectable at low levels in these environmental samples within a bTB-infected area, and the authors suggested that inhalation of contaminated bioaerosols, or drinking contaminated water, might explain at least some cattle infections. The French team has developed a range of methods to evaluate rational interventions.

#### 7.1.3. Nonselective Molecular Surveillance: Whole-Genome-Based Approaches

An alternative to the more straightforward quantitative or endpoint molecular detection methods described above is to apply whole-genome sequencing-based methods to environmental matrices. There is an increasing appreciation of the importance of complex microbiomes in the health of humans, animals, and the environment. Microbiomes can now be surveyed in a relatively unbiased fashion using high-throughput genome sequencing protocols and associated bioinformatics pipelines. Rapid developments in whole-genome sequencing chemistry, throughput, and cost now make it feasible to nonselectively index the microorganisms present in clinical or, potentially, environmental samples in unbiased sequencing surveillance [148]. Such metagenomic next-generation sequencing (mNGS) work is in its infancy; there are very few rigorous published examples, especially relating to TB in man and other animals, but same-day, point-of-care detections are becoming feasible [149].

Zhou et al. [150] reported a small human TB study in China, comparing detections by mNGS in a range of clinical samples with the WHO-endorsed GeneXpert TB PCR instrument, classical culture, and clinical outcome. They concluded that mNGS had a similar diagnostic ability of MTBC compared with GeneXpert, which was more sensitive than conventional culture and showed potential for a variety of clinical samples. While this truly disruptive technology is clearly the direction of travel for modernising microbiology, workflows are not trivial and require significant pathogen genomics capability, capacity, and investment, as well as relevant bioinformatics and biostatistics pipelines.

A recent biomedical example [151] used deep shotgun metagenomics on systematic samples taken from a hospital environment. They included quasi-metagenomics protocols using the nanopore long-range genome sequencing platform (MinION, Oxford Nanopore) to index thousands of genomes, phages, and plasmids from such samples in a relatively unbiased way; >60% were novel. They used a combination of direct shotgun metagenomics protocols for sequencing on a short-read Illumina platform and culture enrichment for long-range nanopore sequencing. The study disclosed the spatial and temporal pattern and diversity of microorganisms sampled from various hospital sites, environments, and matrices and established the feasibility of systematic genome-based surveillance to more rationally target resources in infection control.

In a first pilot study of building-dust microbiomes, protocols were developed for microbiome indexing, based on 16S rRNA sequencing, using both short-read technology (Illumina) and long-read technology (MinION). Despite the well-documented, relatively low read accuracy of the MinION, the outputs, in terms of genera and species detected, were remarkably similar, with MinION reporting greater taxonomic resolution [152]. In addition, proof of concept ultradeep microbiome preparation protocols have been described which facilitate the direct genome sequencing of pathogens from clinical samples [153], and refinements and adaptations continue to be made [154]. As above, while the protocols make sense to a molecular microbiologist, they are not trivial and have not been adapted for application directly to the environmental samples that we might include in our proposed studies. That said, these methods are the lead contenders to progress the “modernising microbiology” agenda.

### 7.2. *In Vitro* Simulation of the Environment: Micro- and Mesocosms

A complimentary way to investigate persistence is through the establishment of *in vitro* laboratory-based models of the environment. Laboratory-based approaches are also useful as a means to “fine-tune” sampling and detection methods for use in the real world. Indeed, this was the approach taken by the Warwick group (see above), who spiked samples with known concentrations of live *M*. *bovis* to optimise the performance of their qPCR methods.

Aside from providing a controlled environment in which to optimise detection methods, *in vitro* methods can also help to address more fundamental issues, such as which environmental factors may be facilitating *M*. *bovis* persistence? These approaches are also flexible and able to embrace simple and more complex setups that incorporate more variables and potentially more hosts. It is a methodology that is still in its infancy, however, and relies on the rational application of direct detection methods (see above). The attendant problems with direct detection, be they molecular or culture based, remain (see above). However, the flexibility gained by using the *in vitro* approach is that the researchers gain more direct control over key risk factors and variables that could affect persistence—climate, soil chemistry, bacterial load, pathogen lineage, etc.

Barbier et al. [124] were able to establish relatively simple microcosms of differing soil types, which they inoculated with *M*. *bovis*. They were unable to detect any effect of soil type on persistence, but did note that, at lower temperatures (4°C vs. 22°C), *M*. *bovis* survival was enhanced for up to 150 days. The cooler temperatures and reduced solar UV that dominate in the UK and Irish climates, compared to continental Europe, have been hypothesised to be possible drivers for enhanced environmental survival [5]. Microcosms also present the possibility of not just investigating the impact of abiotic factors on environmental persistence; there is considerable genotypic and phenotypic variation within the *M*. *bovis* Eu1 clonal complex that is fixed in the UK and Ireland. Could the Eu1 clonal complex, or its descendants, have retained pathogenicity but permitted environmental persistence? [5] The experimental microcosm method would be a means of testing such hypotheses, whereby pathogen evolutionary lineage becomes just one more experimental variable to test, as per Barbier et al. [124].

Additionally, incorporating vertebrate, invertebrate, and/or microbial hosts of *M*. *bovis* into established microcosms may be a useful way of assessing their impact on environmental survival. The larger the host, however, will dictate how large the micro- or indeed mesocosm needs to be. At smaller scales, researchers at the University of Surrey have recently investigated the potential role of FLA, specifically the *Dictyostelium* sps amoeba, which are commonly found in soils. The team found that, unlike other bacteria which are ingested/phagocytosed by amoebae and degraded, *M*. *bovis* survived intracellularly, using the same molecular methods of host evasion it deploys so effectively in host macrophages [101]. Furthermore, the bacillus was able to transmit to other amoebae and replicate at ambient/environmental temperature (25°C), something that was unexpected for an organism thought to be adapted to behave optimally in a vertebrate host environment of ∼37°C [101].

In larger-scale mesocosms, one could envisage addition of other hosts such as earthworms, arthropods, and even potentially small mammals (mice, guinea pigs, or ferrets). However, it is still “early days” with these methods. Starting small with a simple, base model that is a good proof of concept is important. From there, complexity could be increased to embrace differing soil types and simulation of differing climatological factors, such as UVA intensity, precipitation, and temperature.

## 8. Conclusion

bTB is currently the most costly, complex, and indeed controversial multihost, endemic zoonosis in the UK and Ireland. While other countries do not have the same degree of a problem with the disease, it is a potent animal health concern as well as a recognized zoonosis and potential barrier to trade. Evidence across multiple international territories supports the existence of a shared, colocalised, and relatively stable epidemic involving infectious livestock and wildlife hosts. The predominance of local risk factors in most classical and molecular epidemiology studies suggests either that intraspecies transmission occurs in all hosts and that some level of interhost transmission occurs or that sympatric hosts are all equally exposed to an infectious environment. Local epidemiology may differ by region and over time, so it would be unwise to generalise or extrapolate without comprehensive surveillance data.

Surprisingly, despite living with this troubling and costly disease for decades and significant investment in surveillance and research, the actual sources of infection and routes of transmission are not well understood. This limits the options for rational intervention to break chains of transmission. Due to well-documented limitations in diagnostic test performance characteristics, much of the epidemic remains unobserved. Most bTB reactors are now asymptomatic. While direct inhalation is the main route for *M*. *tuberculosis* transmission, intriguingly, other MTBC members may favour other indirect transmission routes. Until relatively recently and largely due to the classical pathology of cattle cases, the received wisdom was that bTB was spread by direct inhalation (2-3 m) and exchange of bioaerosols containing droplets laden with bacteria. It has been hypothesised that tuberculosis is mainly a disease of the lymphatic system, the respiratory tract being seen as both the main entry and the exit point for the pathogen. More recently, due to the very limited detections of direct contact between cattle and wildlife disclosed by observational and behavioural studies using cameras, GPS, and proximity loggers, the potential for indirect transmission via a shared, contaminated environment, including fomites, has been revisited. The possibility that classical pulmonary TB can be recapitulated in laboratory animal models by ingestion of contaminated feed is intriguing and merits further research.

Studies suggest how *M*. *bovis* might be deposited by excretion from infected livestock and/or wildlife to contaminate their shared environment, although excretion is thought to be sporadic. Several studies show that *M*. *bovis* is relatively resilient in the environment, where it can infect a range of vertebrate and invertebrate hosts. Environmental persistence depends on the prevailing weather and landscape conditions. The literature contains few, if any, rational suggestions for intervention. Cattle slurry and, to a lesser extent, manure are considered a risk, for which biosecurity advice has been developed. Properly ensiled forage and composted manure are considered less risky.

Culture-dependent methods and culture-independent molecular methods do exist to attempt to demonstrate viable *M*. *bovis* in the farmed environment. While bacterial culture is possible from environmental samples and matrices, it is considered challenging and of low sensitivity. A range of molecular microbiology, nucleic acid amplification-based, direct detection tests have been developed, and their basic performance characteristics have been described; there is not much to separate which methods could be chosen. However, study design, statistical significance of any size effects, sampling intensity, replication, and quality-assurance validation are important study considerations, which have mostly not adequately been met.

Leading research groups have been able to develop experimental simulations of the environment (micro- or mesocosms), which can be seeded with *M*. *bovis* and other vertebrate and invertebrate hosts, and climatic and other variables can be tested more systematically. Bacteriological and molecular detection tests have been developed and deployed in such studies. It might also be possible to deploy the disruptive technology of next-generation DNA sequencing to the nonselective indexing of the microbiome in simulated or natural environmental samples, matrices, and fomites.

It seems plausible that *M*. *bovis* can be shed into and survive in a variety of environmental matrices and may constitute an epidemiological risk. The relative importance of persistent bacilli for environmental transmission, if it occurs to any great degree, remains to be ascertained. In many cases, “the science” undertaken to determine persistence and potential risk dates from a considerable time ago. It is therefore perhaps timely to apply more modern approaches and systematic analyses to this problem.

Further development of this field of study may well help to uncover transmission mechanisms that until now have remained opaque and also reveal the relative importance of an environmental reservoir of infection for disease persistence. Furthermore, refinement of direct detection methodologies and downstream analyses has use beyond the bTB episystem. Other pathogens such as antimicrobial resistant (AMR) bacteria, which are believed to accumulate in the environment, may be indexed using similar approaches [155].

## Data Availability

No data were used to support this study.
